# Literary Notes

**Published:** 1906-05

**Authors:** 


### LITERARY NOTES.

The Quarterly Journal of Inebriety, the official organ of the American Society for the study of alcohol and other narcotics, has changed its publication office to Boston and will be printed hereafter by the Gorham Press, Richard G. Badger, publisher. Dr. T. D. Crothers, of Hartford, will continue to edit it. It will issue, as before, four times a year and its numbers will be called Spring, Summer, Autumn, and Winter. The spring number for 1906 appears in a handsome new dress and is an excellent specimen of the typographic art.

